# Celastrol ameliorates ulcerative colitis by inhibiting HSP90-mediated necroptosis of intestinal epithelial cells

**DOI:** 10.3389/fphar.2026.1866303

**Published:** 2026-08-21

**Authors:** Keyi Lu, Yuanyuan Wang, Yifan Shi, Siqi Liu, Yanna Shao, Zhibin Wang, Erping Xu

**Affiliations:** 1 Collaborative Innovation Center of Research and Development on the Whole Industry Chain of Yu-Yao, Henan University of Chinese Medicine, Zhengzhou, China; 2 Academy of Chinese Medical Sciences, Henan University of Chinese Medicine, Zhengzhou, China; 3 Department of Critical Care Medicine, School of Anesthesiology, Naval Medical University, Shanghai, China

**Keywords:** celastrol, HSP90, intestinal epithelial barrier, necroptosis, ulcerative colitis

## Abstract

**Introduction:**

Ulcerative colitis (UC) is a chronic inflammatory bowel disease characterized by intestinal barrier dysfunction and epithelial cell death. Necroptosis of intestinal epithelial cells (IECs) mediated by the RIPK1/RIPK3/MLKL pathway has emerged as a key driver of UC progression. Celastrol, a bioactive compound from Celastrus wilfordii, has shown anti-inflammatory potential, but its effect on necroptosis in UC remains unclear.

**Methods:**

A dextran sulfate sodium (DSS)-induced UC mouse model and a TSZ-induced HT-29 cell necroptosis model were established. Disease activity, colon length, histology, mucus barrier integrity, and tight junction protein expression were evaluated. Western blotting, immunofluorescence, qRT-PCR, and a cellular thermal shift assay (CESTA) were used to assess necroptosis and potential targeting of HSP90.

**Results:**

Celastrol significantly improved body weight, disease activity index, and colon length, while restoring mucus secretion and tight junction proteins (ZO-1, Occludin, Muc-2). It reduced phosphorylation of RIPK1, RIPK3, and MLKL both in vivo and in vitro, decreased pro-inflammatory cytokines (TNF-α, IL-1β, IL-6), and attenuated TUNEL-positive IEC death. CESTA indicated that celastrol increased HSP90 thermal stability, suggesting HSP90 engagement.

**Discussion:**

Celastrol ameliorates DSS-induced colitis by inhibiting HSP90-mediated necroptosis of intestinal epithelial cells, supporting its potential as a candidate for UC therapy.

## 1 Introduction

Ulcerative colitis (UC) is a chronic inflammatory disorder of the colon and rectum with rising global prevalence. Clinically, UC manifests as weight loss, abdominal pain, persistent diarrhea, and rectal bleeding and is associated with reduced life expectancy and an increased risk of colorectal cancer (Gros and Kaplan, 2023; Le Berre et al., 2023; Voelker, 2024). Existing therapeutic interventions provide only partial symptom relief and carry substantial risks, including gastrointestinal complications, metabolic disorders, hepatotoxicity, and immunogenicity concerns. These treatments are further limited by poor bioavailability and high costs (Clarisse and Beck, 2021; Naganuma et al., 2022; Pudipeddi et al., 2022; Fletcher et al., 2023). Therefore, there is an urgent need for novel pharmacological interventions for UC prevention and management. Intestinal barrier dysfunction is a key factor in activating aberrant immune responses and sustaining the release of pro-inflammatory cytokines such as tumor necrosis factor-α (TNF-α) and interleukin-1β (IL-1β) (Chen et al., 2021). The integrity of the gut barrier depends heavily on the survival of intestinal epithelial cells (IECs), and their abnormal death can worsen UC. Necroptosis, a regulated form of necrosis (Patankar and Becker, 2020), is a central mechanism of IEC death, mediated by the receptor-interacting protein kinase 1 (RIPK1), receptor-interacting protein kinase 3 (RIPK3), and mixed lineage kinase domain-like pseudokinase (MLKL) signaling pathways (Tao et al., 2023). This process is triggered by prolonged activation of pro-inflammatory cytokines and TNF-α alongside caspase-8 inhibition (Newton, 2015). The formation of the necrosome complex is triggered by the recruitment and activation of RIPK3 following RIPK1 phosphorylation by TNF-α. Subsequently, RIPK3 phosphorylates MLKL, leading to MLKL oligomerization and translocation to the plasma membrane. This results in cellular membrane rupture, the formation of MLKL pores, and the release of damage-associated molecular patterns (DAMPs) (Eldesoqui et al., 2025).

The production of pro-inflammatory cytokines degrades the mucus barrier, increases intestinal permeability, and activates innate immune signaling, which promotes further release of pro-inflammatory cytokines, such as TNF-α and IL-1β, and Th1/Th17 polarization, thereby sustaining inflammation (Park and Cheon, 2021). Necroptosis-induced IEC cell death not only compromises intestinal barrier integrity but also recruits and activates additional immune cells via DAMPs, thereby creating a self-perpetuating cycle of necroptosis and inflammation (Cesaro et al., 2010; Wang et al., 2019). This process also impacts membrane permeability by altering tight junction proteins, including E-cadherin, occludin, and ZO-1 (Wang et al., 2024). Targeting necroptosis with RIPK1 inhibitors such as necrostatin-1 (Nec-1) significantly reduced intestinal inflammation *in vitro* and in intestinal explants cultured from individuals with inflammatory bowel disease (Negroni et al., 2017). Similarly, SZ-15 mitigated dextran sulfate sodium (DSS)-induced UC *in vivo* by reducing colonic mRNA levels of TNF-α, IL-1β, IL-22, and IL-6 (Zeng et al., 2022). Research also indicates that the RIPK3 inhibitor GSK'872 reduced the severity of experimental colitis by suppressing necroptosis and inflammation (Lee et al., 2020). Pristimerin’s effectiveness in UC treatment could also be attributed to its ability to inhibit necroptosis, which enhances intestinal barrier integrity (Liu et al., 2025). Consequently, inhibiting the RIPK1/RIPK3/MLKL necroptotic signaling pathway may improve intestinal barrier function and hinder UC progression.

Notably, although celastrol has been widely reported as an HSP90 inhibitor in inflammation, cancer, and inflammatory bowel disease, most previous studies have focused primarily on its anti-inflammatory or pro-apoptotic properties. In contrast, the role of necroptosis in the pathogenesis and tissue injury of UC has only recently gained attention, and whether celastrol exerts therapeutic effects in UC through modulation of this pathway remains insufficiently explored. Therefore, rather than simply reconfirming the established HSP90-inhibitory activity of celastrol, the present study adopts necroptosis as a central mechanistic axis and integrates target enrichment analysis to investigate the therapeutic potential of celastrol in UC. This study aimed to investigate the molecular mechanism by which Celastrol (CSR) inhibits necroptosis in an UC model, with a specific focus on whether its protective effects HSP90. To investigate the effects of celastrol on IEC necroptosis, we employed a DSS-induced UC model and TNF-α, Smac mimetic, and z-VAD-fmk (TSZ)-induced HT-29 models to evaluate its impact on intestinal barrier function. Target analysis revealed that heat shock protein 90 (HSP90) may mediate celastrol’s protective effect against UC by inhibiting necroptosis. This hypothesis is corroborated by *in vivo* and *in vitro* experiments. This study provides experimental evidence supporting celastrol as a promising therapeutic candidate for UC, potentially by targeting HSP90 to inhibit the necroptosis pathway.

## 2 Materials and methods

### 2.1 Bioinformatics

Celastrol’s possible targets were obtained from the GeneCards database (https://www.genecards.org/), whereas targets associated with UC were obtained from the Open Targets platform (www.opentargets.org) using ‘Ulcerative colitis’ as the search query. Targets related to the necroptosis pathway were identified via GeneCards. All retrieved targets were subsequently imported into the Venn online tool (https://bioinfogp.cnb.csic.es/tools/venny/) to generate Venn diagrams and ascertain overlapping targets among celastrol, UC, and necroptosis pathways. The STRING database was used to perform protein–protein interaction (PPI) analysis on the identified common targets, with the organism set to *Homo sapiens* (https://cn.string-db.org). The obtained PPI data were handled after downloading by employing R (version 4.5.0), including the dplyr and openxlsx packages. Finally, the data were visualized as a PPI network diagram using Cytoscape software (version 3.10.1).

### 2.2 Reagents

Celastrol, with a purity of 99.64% (CAS: 34,157–83-0; HY-13067), was procured from MedChemExpress. The key reagents utilized in this study included GAPDH (Abcam, ab181602) as an internal control, and HRP-conjugated secondary antibodies, specifically goat anti-rabbit IgG (Absin, abs20040) and goat anti-mouse IgG (ABclonal, AS003). Additionally, Human-TNF-α (Novoprotein, C008), z-VAD-fmk (Topscience, T6013) and SM-164 Hydrochloride (MedChemExpress, HY-15989 A) were used in the research. Primary antibodies against RIPK1 (3493 S), phospho-RIPK1 (65746 S), EGF Receptor (4267 T), and HSP70 (4872 T) were used (Cell Signaling Technology). Antibodies against RIPK3 (Abcam, ab209384), phospho-RIPK3, MLKL (ab184718), and phospho-MLKL (ab187091) were obtained from Abcam. Dextran sulfate sodium (DSS; MW: 36,000–50,000) was acquired from Coolaber (CD4421), and Necrostatin-1 was bought from Selleck (S8037). Additional phospho-RIPK1 was provided by Biolind (BX60008), while phospho-RIPK3 (Abcam, ab195117) and RIPK3 polyclonal antibody (Wuhan Sanying, 17563-1-AP) were also employed. MLKL (66675-1-Ig) and phospho-MLKL (Biosynthesis, bsm-54104 R) were included in the study, alongside tight junction proteins Occludin (Servicebio, GB111401) and ZO-1 (Servicebio, GB111402). Anti-HSP90 aerfa/beta antibody (HY-P80714) and tanespimycin (HY-10211) were bought from MedChemExpress. Matrine (T2870) was sourced from the TargetMol. NP-40 Lysis Buffer (P1103 F) was bought from Beyotime. All reagents were utilized in accordance with the manufacturers’ protocols.

### 2.3 Cell culture

Procell Life Science and Technology Co., Ltd. (Wuhan, China) supplied the HT-29 cell line (CL-0118) and was authenticated by STR profiling, which showed a 100% match with the reference data in the Cellosaurus database. The cells were grown in a medium made up of McCoy’s 5 A medium (Procell, CM-0118) with an addition of 10% fetal bovine serum (Procell, 164,210–50) and 1% penicillin/streptomycin (100 U/mL; Gibco, 15,140–122). All cells were maintained at 37 °C in a humid environment with 5% CO_2_.

### 2.4 Cell viability assay

In 96-well plates, HT-29 cells were seeded at a concentration of 10^4 cells per well and incubated for 12 h. Celastrol was dissolved in a medium with and 20 μM Z-VAD-FMK and 10 nM SM-164, achieving final concentrations between 0.125 and 4.0 μM. After the first 12 h of culture, the medium was removed, and the cells were exposed to the drug formulations for 30 min. Following exposure to 20 ng/mL human TNF-α, the TSZ protocol, which includes human TNF-α, SM-164, and Z-VAD-FMK was finalized. Subsequently, the cells underwent incubation for an extra 12 h. The CellTiter-Lumi™ Luminescent Assay Kit (Beyotime, C0056 S) was employed to measure cellular viability.

### 2.5 Double staining of live/dead cells

Following plating in 96-well plates at 1 × 10^4^ cells/well, HT-29 cells were cultured for 12 h. After modeling and intervention with 4 μM celastrol, the supernatant was discarded, Calcein-AM/PI working solution was added both at a ratio of 1:1,000. The cells were then incubated at 37 °C in the dark for 30 min. Cell viability was observed under a microscope (Nikon, Tokyo, Japan), with red fluorescence indicating dead cells and green fluorescence representing live cells.

### 2.6 Assessment of lactate dehydrogenase (LDH) release

Following plating in 96-well plates at 1 × 10^4^ cells/well, HT-29 cells were cultured for 12 h. After modeling and intervention with 4 μM celastrol, the supernatant was collected. Detecting the activity of LDH released into cell culture supernatants using the actate dehydrogenase assay kit (Nanjing jiancheng, A020-2–2) was assigned to analyze necroptosis.

### 2.7 Western blotting

Subsequent to being plated in 6-well plates at a density of 1 × 10^6^ cells per well, HT-29 cells were incubated for 12 h. Treated with TSZ and different concentrations (4 μM, 2μM, and 1 μM) of celastrol for varying durations (0–6 h), cell lysis was carried out for 30 min employing RIPA lysis buffer provided by Cowin Biotech (Jiangsu, China). The supernatant, obtained by centrifugation at 12,000 × g and 4 °C for 10 min, was subjected to protein concentration measurement using a BCA assay kit (Cowin Biotech, Jiangsu, China).10% SDS-PAGE and PVDF membrane (Millipore, USA) was used to separate and transferred the boiled protein samples. 5% BSA is used to block membrane for 2 h. Then washed membrane once with PBST, and incubated overnight at 4 °C with primary antibodies (diluted at 1:1,000). Subsequent to 30 min of rewarming, the membrane underwent three washes in PBST, each lasting 5 min. After incubating the membrane with HRP-conjugated secondary antibodies for an hour, it was washed three times using PBST. Finally, ECL chemiluminescent substrate (Tanon, China) was added, and the membrane was scanned using an imaging system (Bio-Rad, USA) and analyzed using ImageJ software.

### 2.8 Animals

Forty male C57BL/6 J mice (6–7 weeks old) were sourced from Liaoning Changsheng Biotechnology Co., Ltd (China) and maintained under standard conditions (23 °C–25 °C, 50%–60% humidity, 12 h light/dark cycle) with free access to food and water. This study was conducted in compliance with the ARRIVE guidelines and was approved by the Animal Ethics Committee of Henan University of Chinese Medicine (Approval No. DWLLGZR202508058).

### 2.9 Mice model of DSS-induced UC

The mice underwent a 7-day adaptation phase before being randomly divided into two groups: a control group (CON) with autoclaved water and a model group treated with 2.5% DSS in their drinking water for a week to induce UC. On day 7 post-DSS induction, the model group was further randomized into four treatment subgroups (n = 8/group): the DSS-treated model group (DSS), a high-dose celastrol group (Saber et al., 2020) (Celastrol-H, 1 mg/kg), a low-dose celastrol group (Celastrol-L, 0.5 mg/kg), and a Nec-1 group (Geng et al., 2017; Zhong et al., 2023) (5 mg/kg). CSR was 5% DMSO, and 95% saline. The vehicle used for Nec-1 was5% DMSO, and 95% saline. The DSS group resumed normal diet and water, while the treatment groups began daily administration post-modeling: celastrol and Nec-1 were given via intraperitoneal injection, for five consecutive days. On the 12th day, all the animals were euthanized and their colon tissues were collected.

### 2.10 Disease activity index (DAI)

The Disease Activity Index (DAI) was determined by calculating the total points from three key factors. Initially, weight reduction was evaluated on a scale where a 1%–5% decrease received one point, a 5%–10% decline was allotted two points, a 10%–20% loss earned three points, and a decrease exceeding 20% was awarded four points. Next, fecal hemorrhage was graded as follows: 0 points for no occult blood detection, one point for a faint positive result, two points for a moderate positive reading, three points for a strong positive indication, and four points for obvious bleeding. Lastly, stool firmness was assessed with a scoring system: 0 points for typical consistency, one point for softened stools, two points for fluid and viscous feces, three points for watery stools that stick to the anal area, and four points for diarrheal conditions. Furthermore, daily observations were made for body mass, coat appearance, behavioral state, and stool texture. DAI scoring criteria are listed in Table 1.

**TABLE 1:** Disease activity index (DAI) scoring criteria.

Score	Weight loss (%)	Stool consistency	Rectal bleeding
0	0	Normal	Negative (−)
1	0–5	Intermediate	Intermediate
2	5–10	Loose stool	Occult blood (+)
3	10–15	Intermediate	Intermediate
4	≥15	Diarrhea	Gross bleeding

### 2.11 Hematoxylin and eosin (H&E) staining

Mouse tissues including heart, liver, lung, spleen, kidney, colon, were fixed in 4% paraformaldehyde, paraffin-embedded, and sectioned. The sections were subsequently stained using H&E, which included deparaffinization, rehydration, hematoxylin staining (3–5 min), bluing, eosin counterstaining (15 s), and dehydration before mounting and microscopic examination.

### 2.12 Alcian blue-periodic acid schiff (AB-PAS) staining

The paraffin sections were dewaxed and rehydrated, then stained with Alcian blue solution for 10–20 min followed by three 1–2 min washes with distilled water. After oxidation treatment for 5 min, the sections were rinsed with tap water and washed twice with distilled water. Schiff’s reagent staining was performed for 10–20 min, followed by 10 min of running water rinse. Nuclear staining with hematoxylin was conducted for 1–2 min, washed, and differentiated in acid solution for 2–5 s. Sections were blued in Scott’s solution for 3 min and washed for 3 min. Dehydration was carried out through graded alcohols (75%, 95%, and absolute ethanol, 1 min each) and cleared in xylene (three changes, 1–2 min each) before mounting with neutral balsam. For PAS staining, rehydrated sections were stained with solution B for 10–15 min, then treated with solution A for 25–30 min under light-protected conditions. After washing, sections were stained with solution C for 30 s, blued in ammonia water, dehydrated, mounted, and examined microscopically for image acquisition and analysis.

### 2.13 Immunohistochemistry

The paraffin-embedded colon tissue sections were first incubated at 78 °C for 30 min, Subsequently, wax elimination was conducted with xylene, and hydration restoration was accomplished by progressing through an ethanol gradient of sequentially reduced concentrations (90%, 80%, and 70%). After quenching endogenous peroxidase activity with 3% H_2_O_2_ for 10 min, the sections were washed twice with PBS (5 min each time). Primary antibody incubation: Occludin (Servicebio, GB111401), ZO-1 (Servicebio, GB111402), MUC-2 (Servicebio, GB11344), HSP90 (Servicebio, GB12284) (diluted at 1:1,000) was performed at 4 °C overnight, followed by three PBS washes (5 min each time). Subsequently, the sections were incubated with species-matched HRP-conjugated secondary antibodies at room temperature for 50 min protected from light, then washed three times with PBS (5 min each time). Freshly prepared DAB substrate was applied for color development under microscopic monitoring until brown-yellow positive signals appeared, with the reaction terminated by rinsing under running tap water. Finally, the stained sections were examined microscopically for image acquisition and analysis.

### 2.14 Immunofluorescence assay

First deparaffinized and rehydrated Paraffin-embedded intestinal tissue sections for immunofluorescence staining. After antigen retrieval, sections were blocked with 5% BSA for 30 min and then incubated with primary antibodies at 4 °C overnight. Following four washes with PBS, the sections were incubated in the dark for 1 h with the corresponding secondary antibodies: goat anti-rat IgG (GB21303, Servicebio, 1:200) or goat anti-mouse IgG (GB22301, Servicebio, 1:200). Nuclei were then counterstained with DAPI (G1012, Servicebio) for 10 min at room temperature. After final PBS washes, the sections were mounted with anti-fade medium and imaged using a confocal microscope.

### 2.15 Quantitative real-time polymerase chain reaction (qRT-PCR)

For qRT-PCR analysis, total RNA was extracted from mice intestinal tissue by Trizol reagent and reverse-transcribed into cDNA using a reverse transcription kit (E047-01B, Novoprotein). QPCR was then performed using SYBR qPCR SuperMix Plus (E096-01A, Novoprotein) for RNA level analysis. For RNA detection, synthetic RNAs (Sangon Biotech, Shanghai, China) were used. First-strand cDNA was synthesized from RNA using RNA-specific reverse transcription primers (Sangon), followed by quantitative analysis using a cDNA qPCR kit (SYBR Green method, Sangon). The primer sequences are listed in Table 2.

**TABLE 2:** Primer sequence.

Name of primer	Sequences
m-Gapdh-F	AGG​TCG​GTG​TGA​ACG​GAT​TTG
m-Gapdh-R	TGT​AGA​CCA​TGT​AGT​TGA​GGT​CA
m-IL-1b-f	ATC​TTT​TGG​GGT​CCG​TCA​ACT
m-IL-1b-R	GCA​ACT​GTT​CCT​GAA​CTC​AAC​T
m-IL-6-F	TAG​TCC​TTC​CTA​CCC​CAA​TTT​CC
m-IL-6-R	TTG​GTC​CTT​AGC​CAC​TCC​TTC
m-TNF-α -F	ACA​AGG​CTG​CCC​CGA​CTA​C
m-TNF-α -R	TGG​GCT​CAT​ACC​AGG​GTT​TG

### 2.16 Cell thermal shift assay (CESTA)

Total proteins were extracted from cells using NP40 lysis buffer. Protein concentrations were quantified using a BCA assay kit and adjusted to 4 μg/μL with lysis buffer. For the CESTA assay, protein samples were divided into a control group and a celastrol-treated group. Each reaction mixture contained 180 μL of protein lysate. Total proteins were extracted and adjusted to 4 μg/μL. For the CESTA assay, lysates were treated with celastrol 100 μM (or DMSO control) and incubated at room temperature for 1.5 h. For the temperature-gradient assay, samples were aliquoted into six PCR tubes and heated at 48, 50, 52, 54, 56, or 58 °C for 3 min. For the concentration-gradient assay, samples treated with 0, 6.25, 12.5, 25, 50, or 100 μM celastrol were heated at a fixed temperature of 58 °C for 3 min. All heated samples were immediately centrifuged at 12,000 rpm for 15 min at 4 °C to precipitate aggregated proteins. Soluble supernatants were collected, denatured by boiling at 100 °C for 5 min in loading buffer, and analyzed by Western blotting. Subsequently, 20 μL of supernatant from each tube was carefully collected, mixed with 5× SDS-PAGE loading buffer, and denatured by boiling at 100 °C for 5 min. The denatured samples were then resolved by SDS-PAGE and analyzed by Western blotting using an anti-HSP90 antibody to assess the thermal stability of the target protein.

### 2.17 Statistical analysis

A one-way ANOVA followed by Tukey’s multiple comparison test was used to analyze the data, considering *P* < 0.05 as statistically significant.

## 3 Results

### 3.1 Celastrol exhibited anti-necroptotic activity in HT-29 cells

**FIGURE 1:**
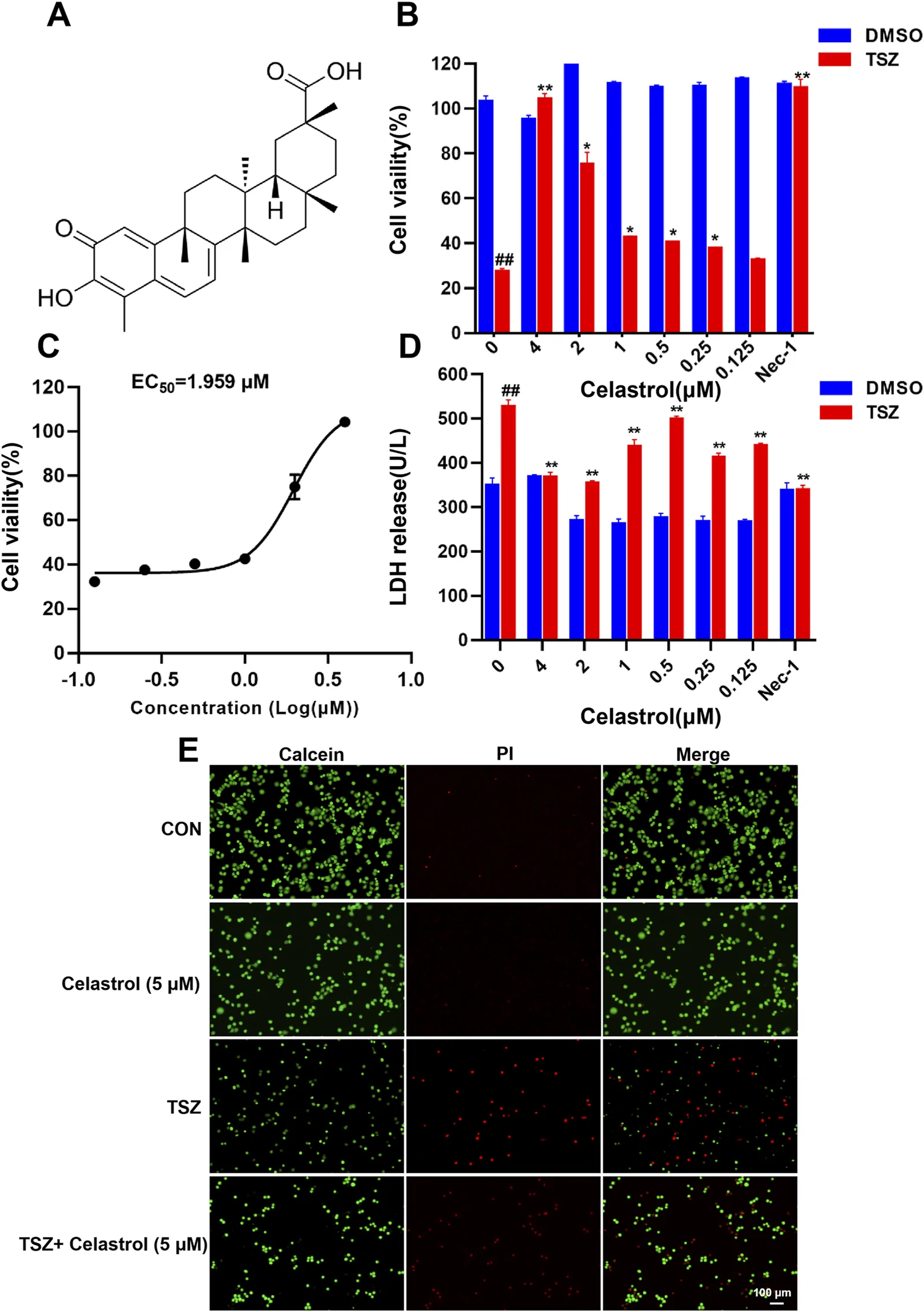
Celastrol exhibits anti-necroptotic activity **(A)** Chemical structure of celastrol **(B)** HT-29 cells were treated with different concentrations of celastrol, followed by TSZ stimulation for 12 h. Cell viability was measured using the CellTiter-Lumi™ luminescent assay **(C)** Half-maximal effective concentration (EC_50_) value calculated based on cell viability results **(D)** HT-29 cells were pre-treated with various concentrations of celastrol before TSZ stimulation for 12 h. LDH release was measured using an LDH assay kit **(E)** Representative calcein-AM/PI staining images of HT-29 cells treated with celastrol for 12 h under different conditions. ^**^ *P* < 0.01, ^*^ *P* < 0.05 versus the TSZ group; ^##^ *P* < 0.01, ^#^ *P* < 0.05 versus the CON group. *N* = 3 per group.

Celastrol exhibited anti-necroptotic activity in murine macrophages (J774 A.1 and BMDMs), human colorectal cancer cells (HT-29), and murine podocytes (MPC-5), as evidenced by reduced phosphorylation of key necroptotic mediators and attenuated cell death *in vitro* and *in vivo* (Liang et al., 2023). However, its effects in HT-29 cells remain unexplored. Accordingly, we assessed the anti-necroptotic effects of celastrol using a TSZ-induced necroptosis model. In the experimental design, the specific RIPK1 kinase inhibitor Nec-1 was used as a positive control, as it potently inhibits TSZ-induced necroptosis by blocking RIPK1 activity, which conclusively demonstrates the engagement of this cell death pathway (Do et al., 2017). The chemical structure of celastrol is displayed in Figure 1A. Celastrol and Nec-1 treatment markedly improved cell viability (Figure 1B), with an EC_50_ value of 1.959 μM (Figure 1C). LDH is a stable cytoplasmic enzyme that is rapidly released into the extracellular space upon the loss of plasma membrane integrity, a key hallmark of cell death. Therefore, measuring LDH activity in the supernatant serves as an indicator of cellular cytotoxicity and necroptosis. LDH assay confirmed celastrol and Nec-1 reduced cell death (Figure 1D). The calcein/PI cell viability/cytotoxicity assay confirmed increased survival. Given that TSZ is a specific and widely accepted inducer of necroptosis, this staining method effectively reports the extent of cellular demise characterized by membrane rupture. In this assay, viable cells with intact membranes and active esterase activity retain intracellular calcein (green fluorescence), whereas PI only enters cells with compromised plasma membranes, binding to nucleic acids and producing red fluorescence, thereby allowing for the quantification of live and dead cell populations. The calcein AM/PI cell viability assay confirmed increased cell survival upon celastrol treatment (Figure 1E), as evidenced by a higher proportion of cells exhibiting green fluorescence (live, calcein AM-positive) and a reduced proportion showing red fluorescence (dead, PI-positive) in the drug-treated group compared to the TSZ group.

### 3.2 Celastrol suppressed the expression and phosphorylation of necroptosis-related proteins

**FIGURE 2:**
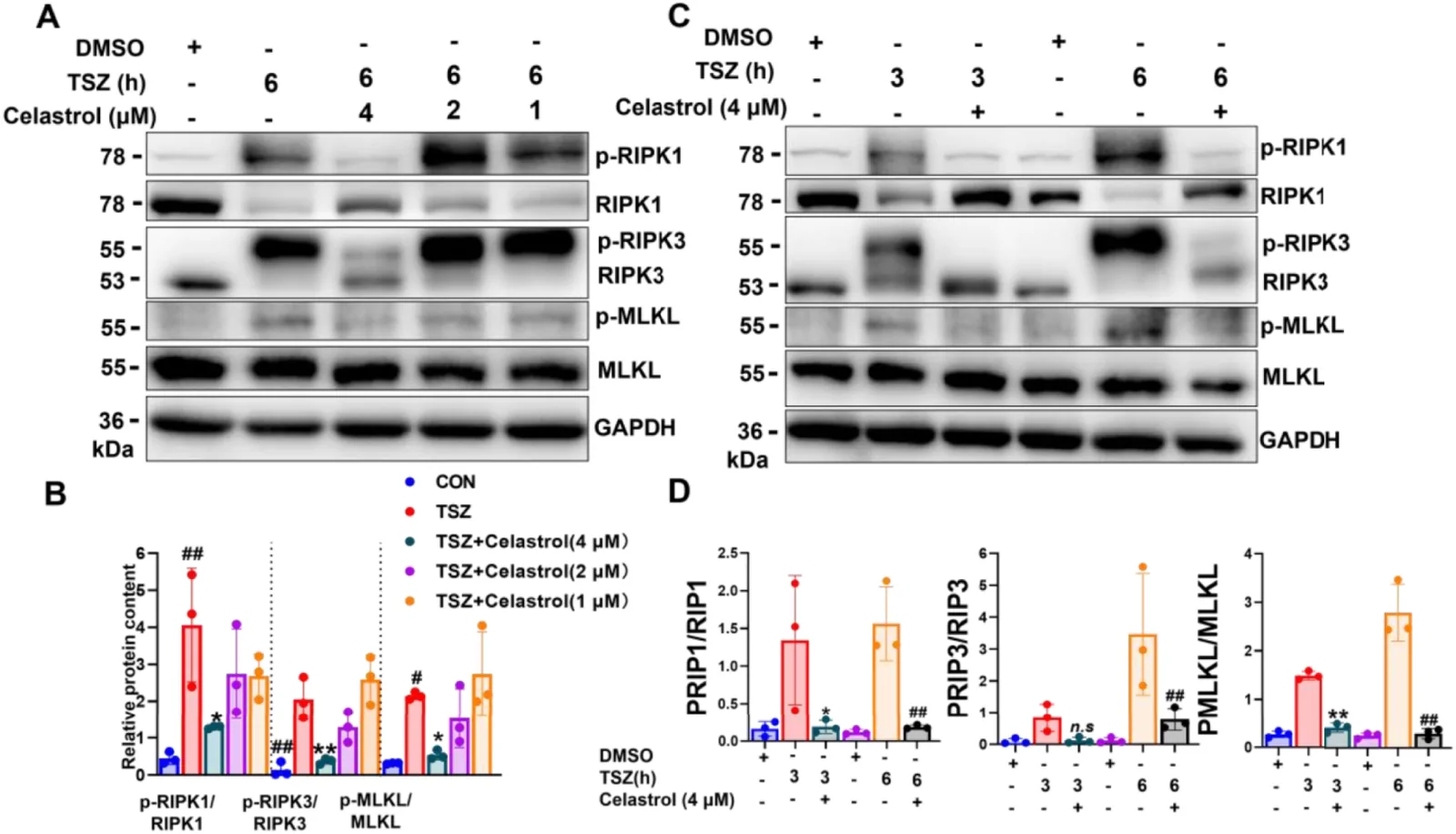
Celastrol suppresses necroptosis by reducing phosphorylation of key regulatory proteins **(A)** HT-29 cells were treated with TSZ and different concentrations of celastrol (4, 2, and 1 μM) for 6 h. All cells were lysed and subjected to immunoblotting with the indicated antibodies **(B)** The quantitative analyses of the representative western blots shown in Figure 2A **(C)** HT-29 cells were incubated with TSZ and celastrol (4 μM) for 0, 3, and 6 h. After incubation, the cells were lysed and analyzed by immunoblotting with the indicated antibodies **(D)** The quantitative analyses of the representative western blots shown in Figure 2C *N* = 3 per group. (Abbreviations: CON, control; p-, phosphorylated; RIPK3, receptor-interacting protein kinase3; RIPK1, receptor-nteracting protein kinase 1; MLKL, mixedlineage kinase domain-like protein.)

The phosphorylation of RIPK1 and RIPK3 promotes necroptosis, while phosphorylated MLKL oligomerizes to form necrosomes, which can compromise membrane integrity and translocate to plasma or organelle membranes (Bertheloot, 2021). We investigated celastrol’s effects on the phosphorylation of MLKL, RIPK3, and RIPK1. Celastrol treatment reduced the phosphorylation levels of these proteins (Figures 2A,B). Furthermore, we observed temporal variations in the phosphorylation levels of these proteins at different time points (Figures 2C,D).

### 3.3 Celastrol alleviated DSS-induced UC in mice

**FIGURE 3:**
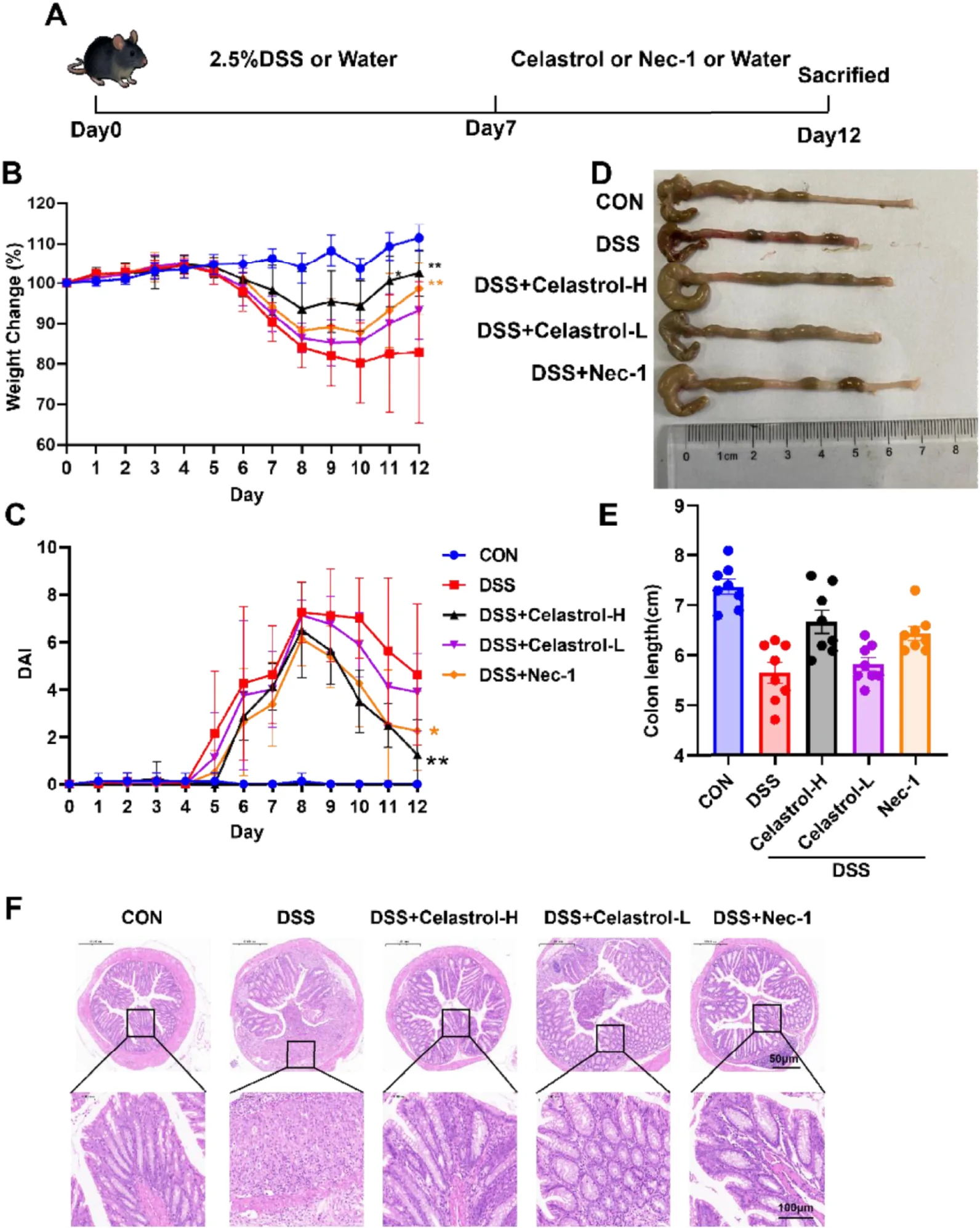
Celastrol alleviates DSS-induced UC in mice **(A)** Schematic illustration of the DSS-induced UC model and experimental drug administration schedule **(B)** Changes in mouse body weight during the experimental period **(C)** Temporal changes in DAI scores **(D)** Sample images of colons from experimental mice **(E)**Temporal changes in stool consistency score. Colon length measurement on Day 12 at sacrifice **(F)** Representative H&E-stained colon tissue sections. *P < 0.05, **P < 0.01 versus the DSS group, ^*^ *P* < 0.05, ^**^ *P* < 0.01 versus the DSS group, ^##^ *P* < 0.01, ^#^ *P* < 0.05 versus the CON group. *N* = 8 per group (Abbreviations: CON, control; Celastrol-H: high-dose celastrol; Celastrol-L: low-dose celastrol; DSS, dextran sulfate sodium; Nec-1, necrostatin-1).

We used a DSS-induced UC model to evaluate the therapeutic effects of celastrol (Figure 3A). Celastrol significantly alleviated UC symptoms, stopping bloody stools, reducing diarrhea and weight loss, and improving animal activity. In the DSS-induced ulcerative colitis animal model, Nec-1 was also utilized as a positive control. It inhibits necroptosis by specifically targeting RIPK1, thereby serving to verify the inhibitory effect of the candidate compound celastrol on necroptosis in intestinal epithelial cells (Liu et al., 2015). The Celastrol -H and Nec-1 group demonstrated a recovery in body weight compared with the DSS group (Figure 3B) and exhibited reduced DAI (Figure 3C). Colon length was restored more effectively in the Celastrol -H group and Nec-1 than in the DSS group (Figures 3D,E). Hematoxylin and eosin (H&E)-stained sections of colon tissue revealed that celastrol and Nec-1 reduced submucosal edema and inflammatory infiltration (Figure 3F).

### 3.4 Celastrol preserved intestinal barrier function in DSS-induced UC mice

**FIGURE 4:**
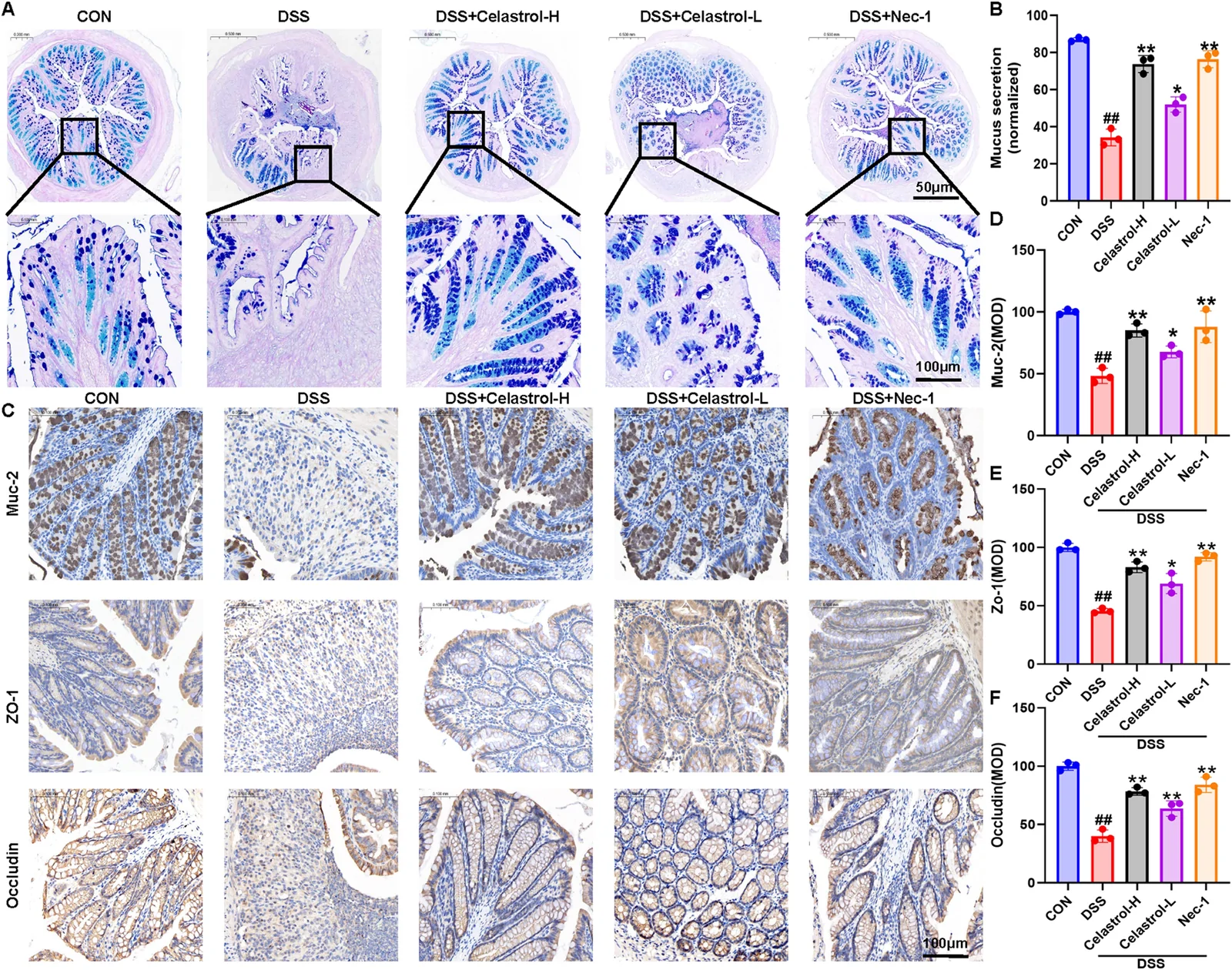
Celastrol preserves the integrity of the colonic mucus barrier in DSS-induced UC mice **(A)** Representative AB-PAS staining images of colonic tissue sections **(B)** Quantitative analysis of AB-PAS staining results **(C)** Representative immunohistochemical staining images of Muc-2, ZO-1, and occludin in colonic tissues **(D–F)** Quantitative analysis of normalized expression levels of Muc-2, ZO-1, and occludin. ^*^ *P* < 0.05, ^**^ *P* < 0.01 versus the DSS group; ^##^ *P* < 0.01, ^#^ *P* < 0.05 versus the CON group. *N* = 3 per group. (Abbreviations: CON, control; Celastrol-H:, high-dose celastrol; Celastrol-L, low-dose celastrol; DSS, dextran sulfate sodium; Muc-2, mucoprotein2; Nec-1, necrostatin-1; Occludin, Occludens-1; ZO-1, Zona Occludens 1.)

Intestinal barrier integrity plays a critical role in UC pathogenesis. Goblet cells secrete mucins, particularly Muc-2, which serve as the primary structural components of the intestinal mucus layer. This mucin barrier forms a critical physical defense system, protecting the intestinal epithelium from pathogenic microorganisms and harmful luminal substances. DSS-induced UC disrupts this barrier, reducing goblet cell numbers and mucin production (Van der Sluis et al., 2006). Results revealed that in the DSS group, inflammatory injury significantly reduced goblet cell numbers, consequently reducing AB-PAS-positive stained areas (Figures 4A,B). Celastrol and Nec-1 treatment restored goblet cell populations and increased AB-PAS-positive stained areas. Immunohistochemical analysis demonstrated that Muc-2 expression, significantly downregulated in DSS mice, was restored by celastrol and Nec-1 treatment (Figures 4C,D).

Tight junctions between IECs are essential for maintaining epithelial integrity, with occludin and ZO-1 serving as key structural proteins (Schwayer et al., 2019). Immunohistochemical analysis revealed that DSS treatment significantly reduced the expression of occludin and ZO-1 compared with the control (CON) group, indicating disruption of the intestinal mucosal barrier. Treatment with celastrol and Nec-1 markedly increased the expression and improved the distribution of occludin and ZO-1 (Figures 4C,E,F). These findings demonstrate that celastrol enhances intestinal barrier function in UC mice by reinforcing both the mechanical and mucous barriers.

### 3.5 Celastrol prevented necroptosis in IECs

**FIGURE 5:**
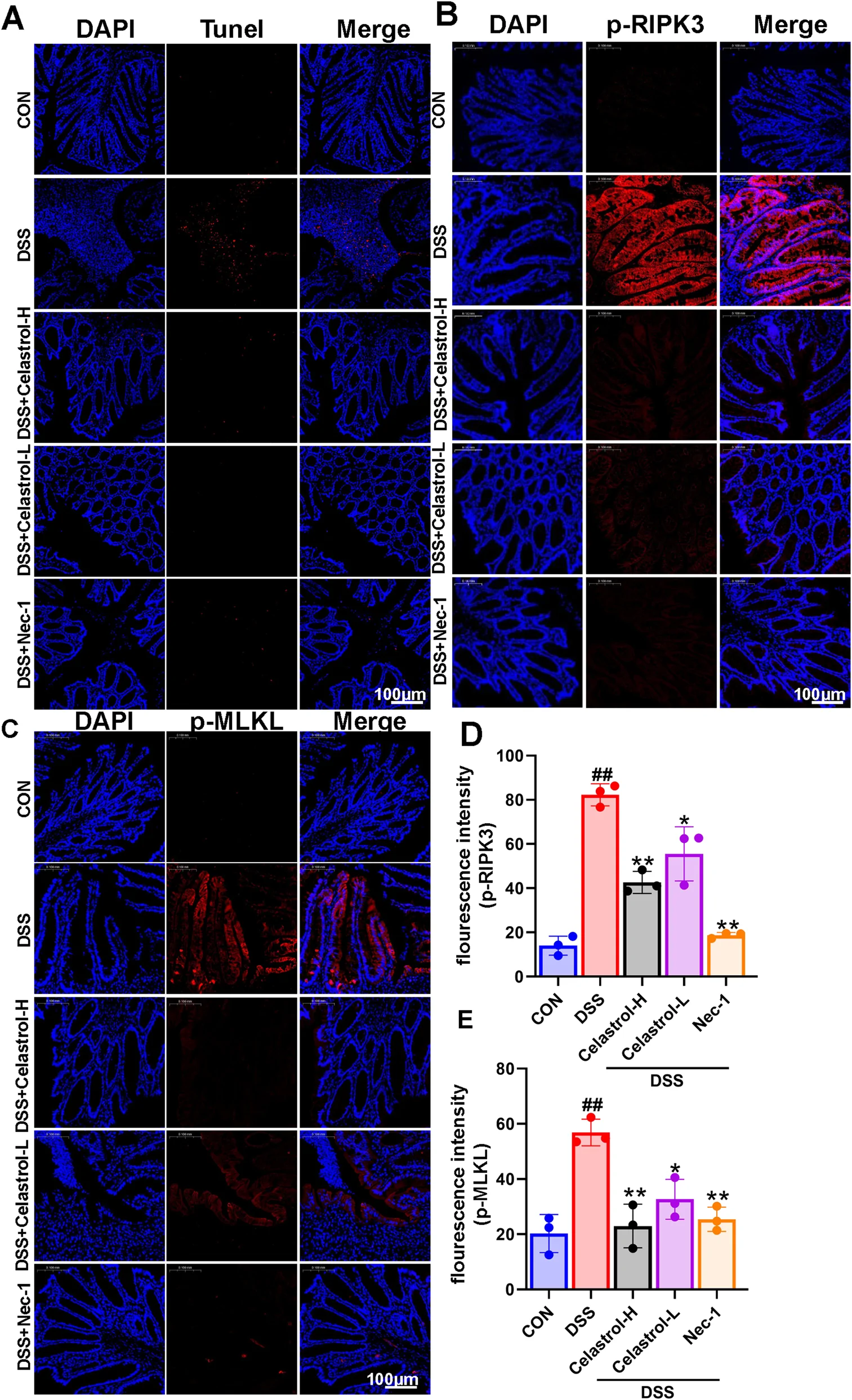
Celastrol protects IECs from necroptosis **(A)** Representative fluorescent TUNEL staining images depicting IECs necroptosis in colonic tissues **(B,C)** Immunofluorescence analysis of the distribution and expression of p-RIPK3 and p-MLKL in IECs **(D,E)** Quantitative analysis of fluorescence intensity for p-RIPK3 and p-MLKL. ^*^ *P* < 0.05, ^**^ *P* < 0.01 versus the DSS group, ^##^ *P* < 0.01, ^#^ *P* < 0.05 versus the CON group. *N* = 3 per group. (Abbreviations: CON, control; Celastrol-H:, high-dose celastrol; Celastrol-L, low-dose celastrol; DSS, dextran sulfate sodium; Nec-1, necrostatin-1; p-, phosphorylated; RIPK3, receptor-interacting protein kinase3; MLKL, mixedlineage kinase domain-like protein.)

TUNEL staining was performed to evaluate cell death in IECs. Necroptosis occurs largely in the villi intestinal epithelium cells. The DSS group exhibited a significant increase in TUNEL-positive cells compared with the CON group, whereas celastrol treatment reduced the number of TUNEL-positive cells. Notably, the Celastrol-H group demonstrated comparable efficacy to the Nec-1 group (Figure 5A). These results indicate that celastrol effectively alleviates IEC necroptosis in DSS-induced UC.

Necroptosis is characterized by the phosphorylation of RIPK3 and MLKL, which is an important pathological event. To evaluate celastrol’s effect on necroptosis in IECs, the levels of phosphorylated RIPK3 and MLKL were examined. Results revealed that DSS treatment increased the phosphorylation levels of MLKL and RIPK3, whereas celastrol reduced their phosphorylation levels. The Celastrol-H group exhibited effects comparable to those observed in the Nec-1 group (Figures 5B–E). These findings suggest that celastrol suppressed necroptosis in the colons of UC mice, thereby strengthening intestinal barrier integrity and contributing to its therapeutic effect.

### 3.6 Celastrol suppressed intestinal inflammation in the intestinal tissues of UC mice

**FIGURE 6:**
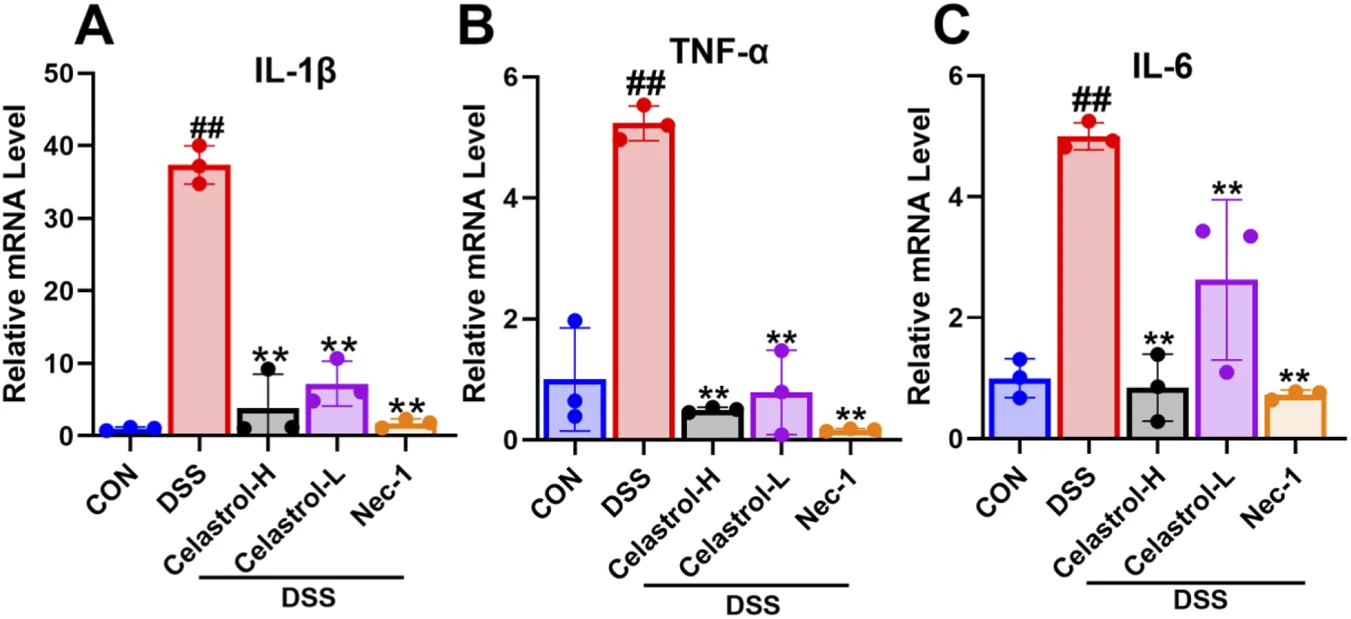
Celastrol alleviates inflammation in UC mice. mRNA expression levels of **(A)** IL-1β **(B)** TNF-α, and **(C)** IL-6. ^*^ *P* < 0.05, ^**^ *P* < 0.01 versus the DSS group; ^##^ *P* < 0.01, ^#^ *P* < 0.05 versus the CON group. *N* = 3 per group. (Abbreviations: CON, control; Celastrol-H, high-dose celastrol; Celastrol-L, low-dose celastrol; DSS, dextran sulfate sodium; Nec-1, necrostatin-1.)

The primary therapeutic objective in UC is to reduce intestinal inflammation and restore intestinal barrier integrity. Therefore, we assessed inflammatory cytokine levels. TNF-α serves as a primary inducer of necroptosis by activating the RIPK1/RIPK3/MLKL pathway (Newton and Manning, 2016), triggering necroptosis, disrupting the intestinal epithelial barrier, and promoting mucosal injury in UC. IL-1β, released upon activation of the NLRP3 inflammasome (Xu and Núñez, 2023), promotes neutrophil infiltration and amplifies inflammatory responses, thereby aggravating intestinal inflammation and necroptosis. IL-6 promotes Th17 cell differentiation and mucosal immune dysregulation through STAT3 signaling while suppressing Treg function, thereby sustaining chronic inflammation and impairing tissue repair in UC (Johnson et al., 2018). Collectively, these cytokines promote a chronic inflammatory microenvironment that contributes to UC progression. Reducing the levels of these inflammatory cytokines is critical for suppressing necroptosis and treating UC. Results revealed that the DSS group exhibited significantly elevated mRNA expression levels of pro-inflammatory cytokines (IL-1β, TNF-α, and IL-6) compared with the CON group. In contrast, treatment with celastrol at high and low doses, as well as Nec-1, significantly reduced the mRNA expression levels of IL-1β, TNF-α, and IL-6 compared with the DSS group (Figures 6A–C).

### 3.7 Prediction of potential targets of celastrol in inhibiting necroptosis in UC

**FIGURE 7:**
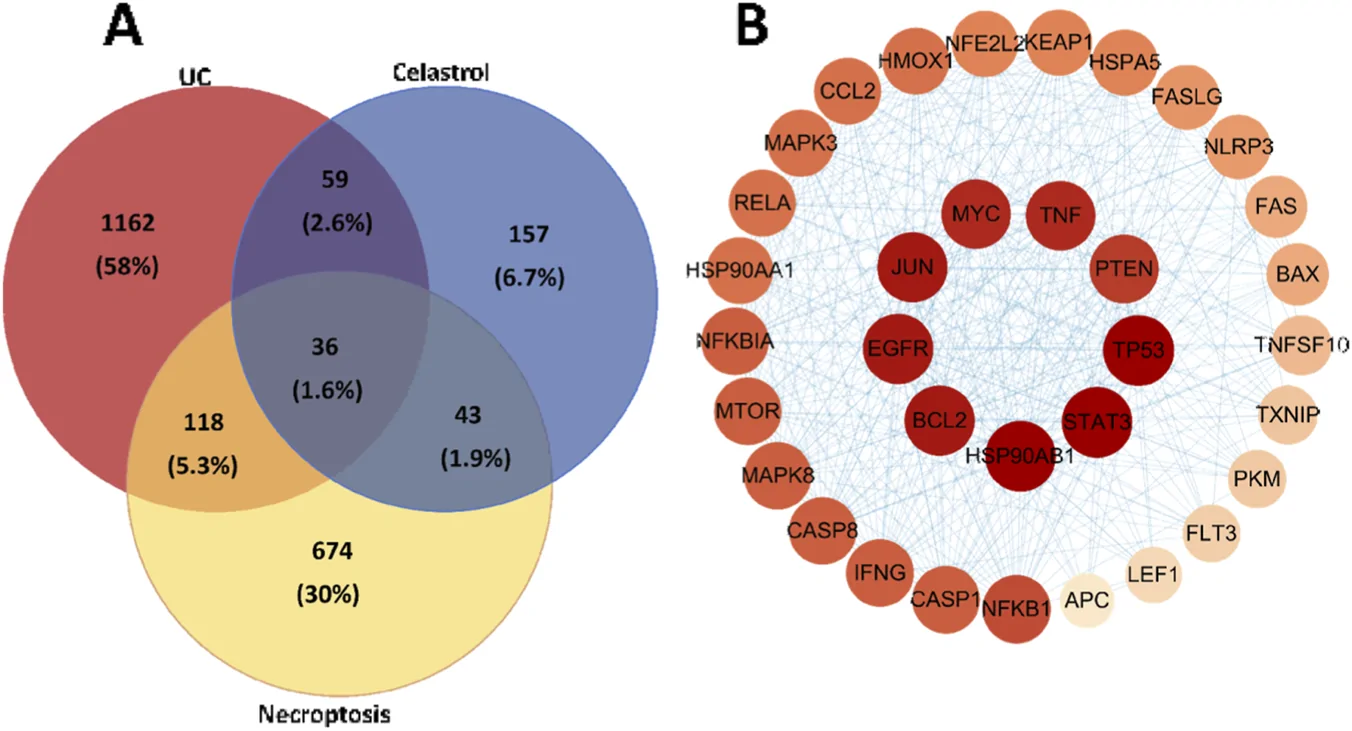
HSP90AB1 is the core component of the celastrol protein–protein interaction (PPI) network **(A)** Venn diagram illustrating overlapping targets among celastrol, necroptosis, and ulcerative colitis **(B)** PPI network of potential therapeutic targets.

To identify the potential targets through which celastrol may inhibit necroptosis in the treatment of UC, we performed an intersection analysis of targets associated with celastrol, UC, and necroptosis. The analysis yielded 36 shared targets across the three datasets (Figure 7A). Subsequent protein–protein interaction (PPI) analysis suggests that HSP90 is a potential target (Figure 7B). We focused on HSP90 for further investigation because it is a known upstream regulator of necroptosis executioners (RIPK1/RIPK3) (Zhou et al., 2024), Furthermore, it has been documented that celastrol functions as an HSP90 inhibitor, which provides a direct mechanistic hypothesis for experimental validation (Aceros et al., 2019; Yang et al., 2025).

### 3.8 Celastrol inhibited IEC necroptosis by targeting HSP90

**FIGURE 8:**
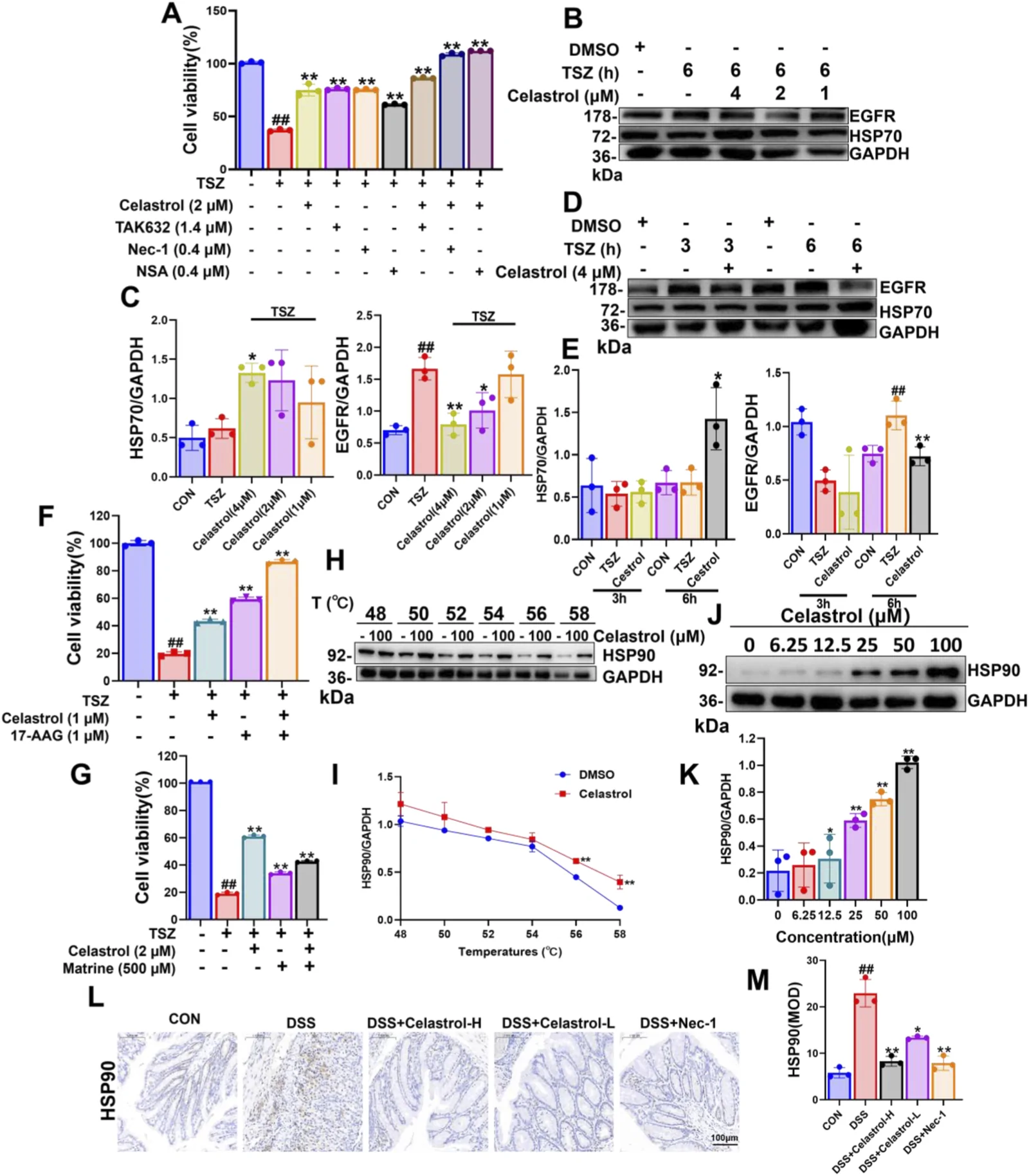
Celastrol suppresses IEC necroptosis by targeting HSP90 **(A)** HT-29 cells were treated with celastrol, TAK632, Nec-1, or NSA alone or in combination, followed by TSZ stimulation for 12 h. Cell viability was assessed using CellTiter-Lumi™ luminescent assay **(B)** HT-29 cells were treated with TSZ and different concentrations of celastrol (4, 2, and 1 μM) for 6 h, followed by cell lysis and immunoblotting with the indicated antibodies **(C)** The quantitative analyses of the representative western blots shown in Figure 8B **(D)** HT-29 cells were treated with TSZ and celastrol (4 μM) for 0, 3, and 6 h and analyzed by immunoblotting using the indicated antibodies **(E)** The quantitative analyses of the representative western blots shown in Figure 8E **(F)** HT-29 cells were treated with celastrol, 17-AAG alone or in combination, followed by TSZ stimulation for 12 h. Cell viability was assessed using CellTiter-Lumi™ luminescent assay **(G)** HT-29 cells were treated with celastrol, matrine alone or in combination, followed by TSZ stimulation for 12 h. Cell viability was assessed using CellTiter-Lumi™ luminescent assay **(H)** Representative Western blots showing HSP90 thermal stability following treatment with 100 μM celastrol or DMSO control across a temperature gradient of 48 °C–58 °C **(I)** The quantitative analyses of the representative western blots shown in Figure 8H. ^*^ *P* < 0.05, ^**^ *P* < 0.01 versus the DMSO group **(J)** Representative Western blots of HSP90 stability at a constant temperature of 58 °C following treatment with increasing concentrations of celastrol (0–100 μM) **(K)** The quantitative analyses of the representative western blots shown in Figure 8J **(L)** Representative immunohistochemical staining images of HSP90 in colon tissues **(M)** Quantitative analysis normalized HSP90 expression. ^*^ *P* < 0.05, ^**^ *P* < 0.01 versus the DSS or TSZ group. ^##^ *P* < 0.01, ^#^ *P* < 0.05 versus the CON group. *N* = 3 per group. (Abbreviations: CON, control; Celastrol-H, high-dose celastrol; Celastrol-L, low-dose celastrol; DSS, dextran sulfate sodium; EGFR, Epidermal Growth Factor Recepto; HSP70, Heat Shock Protein 70; HSP90, Heat Shock Protein 90; Nec-1, necrostatin-1.)

Celastrol is recognized as an HSP90 inhibitor; however, its potential therapeutic role in UC through HSP90-mediated inhibition of necroptosis has not been investigated (Zhang et al., 2009). To determine whether celastrol inhibits necroptosis through HSP90 regulation, we evaluated its effects in combination with necroptosis inhibitors. Results revealed that the efficacy of celastrol did not reduce when co-administered with specific necroptosis inhibitors, including Nec-1, TAK632, and NSA. This finding suggests the possibility that celastrol may operate, at least in part, via a mechanism distinct from, or parallel to, the classical necroptosis pathway mediated by RIPK1, RIPK3, and MLKL (Figure 8A). However, alternative interpretations, such as the use of sub-saturating inhibitor concentrations, cannot be fully excluded, and further studies are warranted to conclusively delineate the precise mechanism. Subsequently, we evaluated the expression levels of EGFR and HSP70, which serve as indicators of HSP90 activity. Celastrol treatment reduced EGFR expression and increased HSP70 expression (Figures 8B–E). To investigate whether the anti-necrotic effect of celastrol was HSP90-dependent, we performed combination assays with HSP90-modulating agents. The canonical HSP90 inhibitor 17-AAG (Konstantinopoulos and Papavassiliou, 2005) restored cell viability, consistent with its reported activity. Celastrol alone also significantly improved viability versus the model group. Notably, co-administration of celastrol and 17-AAG produced an additional increase in cell viability compared with either agent alone (Figure 8F), suggesting that although both engage HSP90, they may act through distinct mechanistic facets rather than simple redundancy. In support of this, co-treatment with Matrine, a reported HSP90 activator (Tanabe et al., 2018), partially counteracted the protective effect of celastrol, reducing cell viability relative to celastrol alone (Figure 8G). To determine whether celastrol directly binds HSP90, we performed a Cellular Thermal Shift Assay. At 100 μM, celastrol increased the thermal stability of HSP90 across 48 °C–58 °C (2 °C intervals), with soluble HSP90 remaining detectable at higher temperatures where the DMSO control was largely precipitated (Figures 8H,I). At a fixed temperature of 58 °C, HSP90 stabilization showed a clear concentration dependence (0–100 μM; Figures 8J,K). *In vivo*, immunohistochemical staining of colonic tissues revealed elevated HSP90 expression in the model group, which was reduced by celastrol treatment (Figures 8L,M). Collectively, these findings suggest that celastrol exerts its therapeutic effects by targeting HSP90 to suppress necroptosis, thereby alleviating UC.

### 3.9 Celastrol exhibited no toxic effects in UC mice

**FIGURE 9:**
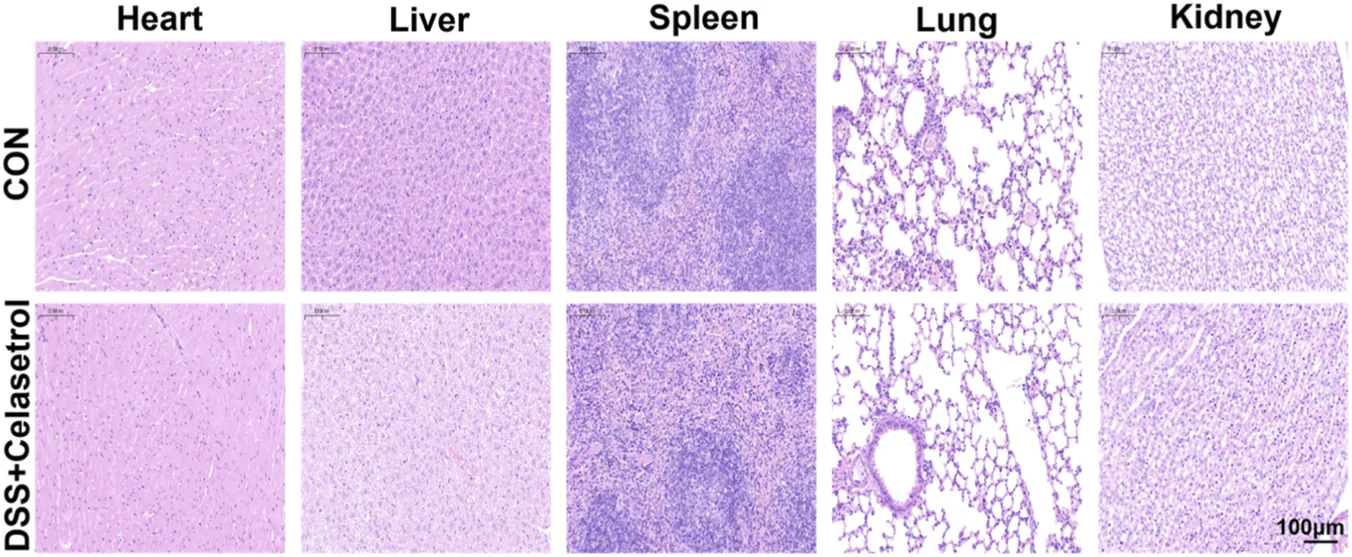
Celastrol exhibits no toxicity in major organs. Representative H&E staining of heart, lung, liver, spleen, and kidney tissues from UC mice treated with celastrol.

Previous studies have reported potential toxicity associated with celastrol (Hou et al., 2020). To evaluate its safety at therapeutic dosages, histopathological examinations of major organs were performed using H&E staining. The hearts, livers, spleens, lungs, and kidneys of UC mice treated with celastrol (1 mg/kg) were examined. Similar to the CON group, the Celastrol-H group demonstrated no signs of toxicity in these organs, indicating that celastrol at a 1 mg/kg dose is safe (Figure 9).

## 4 Discussion

Approximately 8% of individuals with UC develop colorectal cancer, a condition that remains incurable (León-Vega et al., 2025). Although advanced pharmacotherapies, including TNF-α antagonists, vedolizumab, IL-12/23 or specific IL-23 antagonists, and JAK inhibitors, are available, a subset of patients exhibit poor responsiveness and ultimately require surgical intervention (Ananthakrishnan et al., 2024). Traditional Chinese medicine and its natural products have demonstrated favorable safety profiles during long-term use (Hu et al., 2021; Wei et al., 2021). In this study, celastrol significantly alleviated UC symptoms, as evidenced by reduced body weight, reduced DAI, restored colon length, reduced IEC death, reduced production of pro-inflammatory cytokines, and improved intestinal barrier integrity. In addition, celastrol exhibited significant anti-necroptotic activity *in vitro* and *in vivo*. It improved cell viability, LDH content, and cell morphology by reducing the phosphorylation levels of RIPK1, RIPK3, and MLKL in HT-29 cells. Importantly, celastrol administered at the tested therapeutic dosage did not produce detectable toxicity in major organs. These findings highlight the potential of celastrol as a necroptosis inhibitor for UC treatment.

HSP90 is a crucial molecular chaperone that maintains the activity of several components of the necrotic signaling cascade. Studies demonstrating that HSP90 suppression promotes RIPK1 degradation and prevents TNF-induced NF-κB activation suggest that HSP90 interacts with and stabilizes RIPK1 through its co-chaperone CDC37 (Lewis et al., 2000; Chen et al., 2002). This interaction facilitates the recruitment of RIPK1 to the TNF receptor signaling complex and protects it from proteasomal destruction. HSP90 also plays a critical role in stabilizing MLKL, the primary effector of necroptosis. By binding directly to MLKL, HSP90 prevents its ubiquitin-proteasomal degradation and promotes phosphorylation-dependent oligomerization, which is required for pore formation and membrane translocation (Jacobsen et al., 2016; Zhao et al., 2016). HSP90 suppression prevents necroptosis-mediated tissue damage in heart failure (Marunouchi et al., 2021) and cholangiocyte cell death in primary biliary cholangitis (Chen et al., 2022), underscoring the importance of HSP90 regulation in disease contexts. Recent studies have demonstrated that HSP90 inhibitors exert therapeutic effects in UC by alleviating DSS-induced colitis through mechanisms such as suppression of the MAPK pathway (Yuan et al., 2022) and targeting HSP90β to regulate fatty acid synthesis and inhibit NLRP3 inflammasome activation (Yang et al., 2023).

**FIGURE 10:**
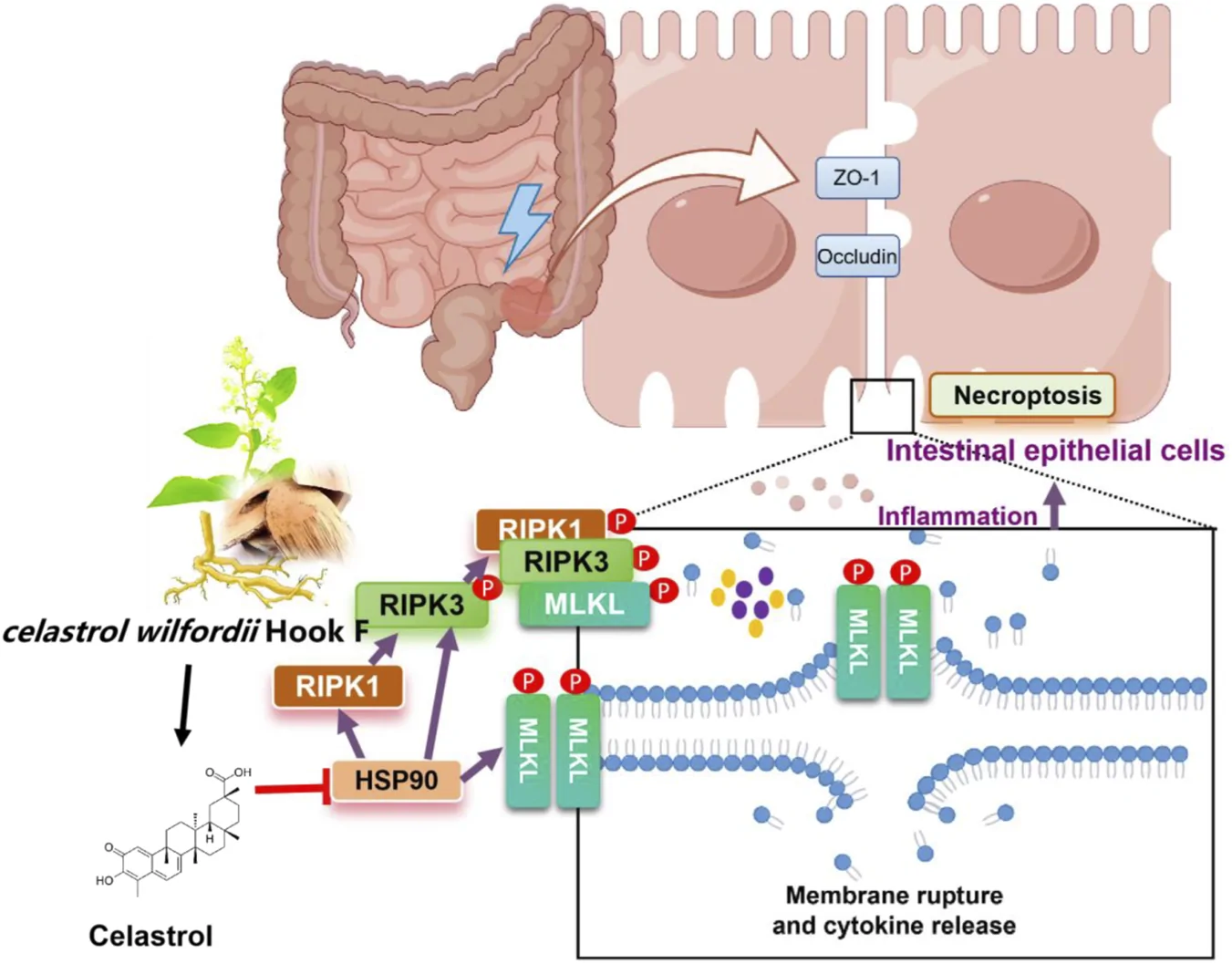
Proposed mechanism by which celastrol protects against UC (Abbreviations: CON, control; DSS, dextran sulfate sodium).

Celastrol is a well-known HSP90 inhibitor and has been reported to improve radiosensitivity in lung cancer cells (Lee et al., 2011) and modulate HSP90 function in treating tibial dysplasia (Nabi et al., 2016). Our experiments using necroptosis inhibitors demonstrated that celastrol, when combined with inhibitors targeting the necroptotic pathway, enhanced the cell activity induced by TSZ. Consistent with its known mechanism, the necroptosis inhibitor Nec-1 alleviated colitis and promoted epithelial repair, confirming that necroptosis is a key driver of pathology in our model. This established the necessary benchmark, thereby supporting that the potent protective effect observed with our candidate compound celastrol likely operates through a related, and potentially superior or multi-target, mechanism of necroptosis inhibition. This observation suggests that celastrol may suppress necroptosis through a mechanism independent of the classical necroptosis pathways. Further bioinformatic analyses indicated that HSP90 is a potential molecular target of celastrol. *In vitro* experiments demonstrated that celastrol reduced HSP90 activity, as reflected by decreased EGFR expression and increased HSP70 levels. Such mechanistic insights will be critical for advancing the translational potential of celastrol in UC treatment. A schematic illustration of the proposed mechanism is presented in Figure 10.

However, given the complex interactions among various cell death pathways, celastrol may also exert effects through additional mechanisms, including pyroptosis, apoptosis, or autophagy. The precise mechanism by which HSP90 modulates the necroptotic signaling cascade, potentially through the regulation of the RIPK3-MLKL axis via client proteins, remains unclear. Future studies employing cell-type-specific knockout models, high-throughput transcriptomic and proteomic methodologies, and molecular interaction assays will be essential to elucidate the precise targets and signaling networks through which celastrol exerts its therapeutic effects. While celastrol is a well-established HSP90 inhibitor with documented anti-inflammatory and anti-apoptotic effects in IBD models, prior studies have largely overlooked its role in modulating necroptosis, a key driver of epithelial injury in UC. By integrating target enrichment analysis, we identified HSP90 as a candidate node linking celastrol to necroptosis suppression in UC. Although this target has been previously reported, our study provides novel insights into its involvement in necroptosis-mediated UC therapy, though further validation of additional regulatory mechanisms is warranted. A limitation of the current study is the lack of genetic evidence to definitively establish HSP90 as the direct molecular target mediating celastrol’s therapeutic effects in UC. While bioinformatics analyses and pharmacological data suggest HSP90 is a key node, causality requires validation via genetic approaches, such as HSP90 knockdown or overexpression in intestinal epithelial cells. Future studies employing these techniques are essential to confirm the direct involvement of the HSP90-necroptosis axis in UC. Another recognized limitation of the current study is that the *in vivo* safety assessment relied primarily on histopathological examination and gross clinical observations. While no overt organ toxicity was detected via H&E staining at the therapeutic dose, comprehensive serum biochemistry (e.g., ALT, AST, BUN, Cr) and complete blood count analyses were not performed. Functional biomarkers provide a more sensitive assessment of hepatorenal status and may detect subtle perturbations preceding morphological changes. Therefore, while this proof-of-concept study establishes the therapeutic potential of celastrol in UC, future dedicated preclinical toxicology studies incorporating dose-escalation schemes, expanded histopathology, and full serum chemistry panels are warranted to comprehensively define its safety window prior to translational development.

## 5 Conclusion

In this study, celastrol alleviated body weight loss, reduced the DAI, and restored colon length in UC mice. Moreover, celastrol attenuated IEC death and suppressed the phosphorylation of key necroptotic mediators, including RIPK1, RIPK3, and MLKL. Our *in vivo* experiments confirmed that celastrol decreased the mRNA expression of key pro-inflammatory cytokines, specifically IL-1β, TNF-α, and IL-6. Collectively, these findings indicate that celastrol exerts protective effects in UC by suppressing necroptosis and inflammatory response. Further *in vitro* and *in vivo* experiments indicate that the therapeutic efficacy of celastrol may be associated with HSP90 downregulation. In conclusion, our study suggests that the ameliorative effect of celastrol on UC is related to the inhibition of necroptosis, in which HSP90 might be implicated. These results contribute to the mechanistic understanding of celastrol and highlight a potential therapeutic axis for intestinal inflammation.

## Data Availability

The raw data supporting the conclusions of this article will be made available by the authors, without undue reservation.
