# Correction: Celastrol ameliorates ulcerative colitis by inhibiting HSP90-mediated necroptosis of intestinal epithelial cells

**DOI:** 10.3389/fphar.2026.1974947

**Published:** 2026-09-21

**Authors:** Keyi Lu, Yuanyuan Wang, Yifan Shi, Siqi Liu, Yanna Shao, Zhibin Wang, Erping Xu

**Affiliations:** 1 Collaborative Innovation Center of Research and Development on the Whole Industry Chain of Yu-Yao, Henan University of Chinese Medicine, Zhengzhou, China; 2 Academy of Chinese Medical Sciences, Henan University of Chinese Medicine, Zhengzhou, China; 3 Department of Critical Care Medicine, School of Anesthesiology, Naval Medical University, Shanghai, China

**Keywords:** celastrol, Hsp90, intestinal epithelial barrier, necroptosis, ulcerative colitis

There was a mistake in Figure 1 as published. During the preparation of the figure, an incorrect merged image was inadvertently used for the control (con) group, which resulted in the control group panel not accurately representing the intended experimental condition. The corrected Figure 1 appears below.

**FIGURE 1:**
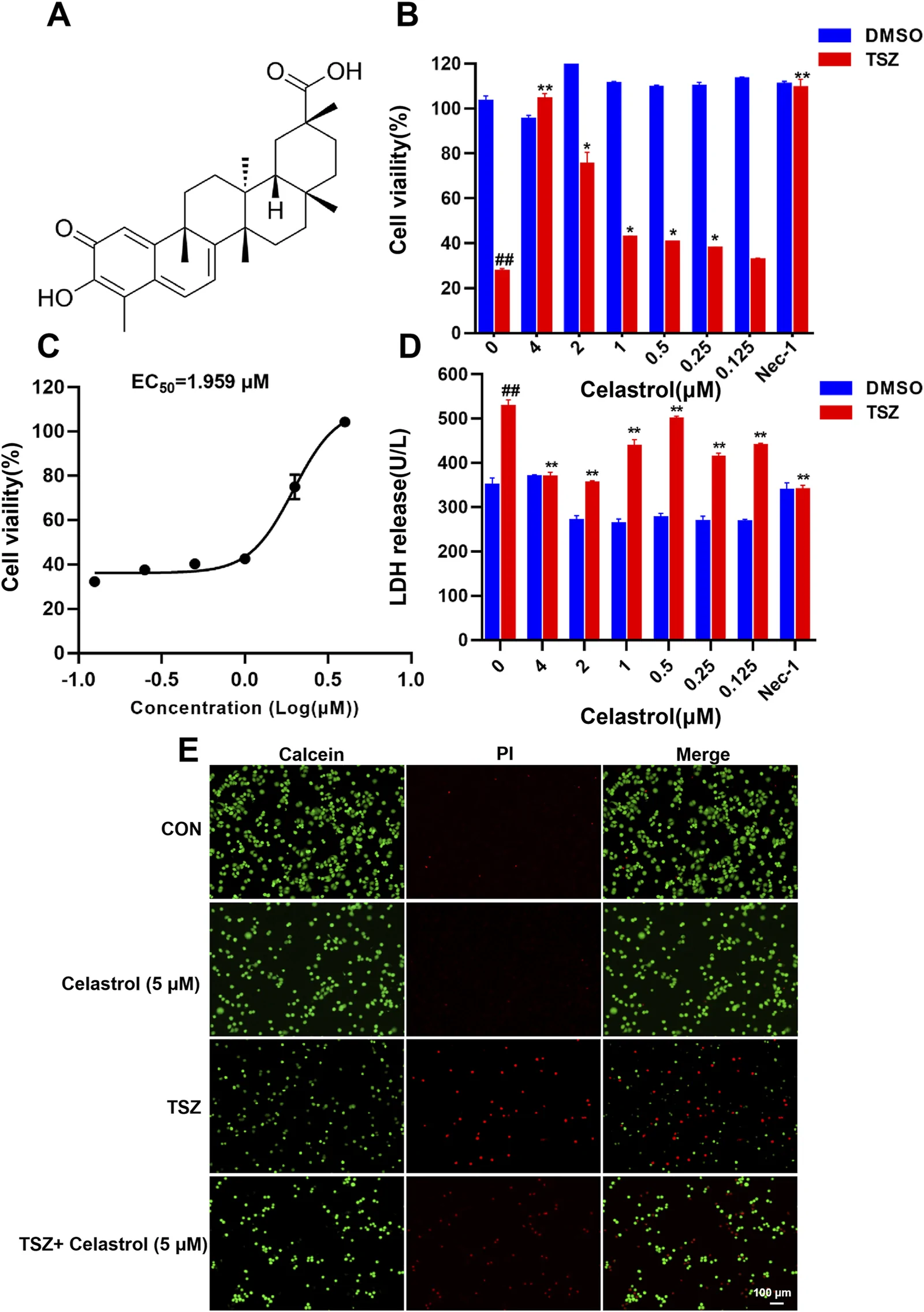
Celastrol exhibits anti-necroptotic activity **(A)** Chemical structure of celastrol **(B)** HT-29 cells were treated with different concentrations of celastrol, followed by TSZ stimulation for 12 h. Cell viability was measured using the CellTiter-Lumi™ luminescent assay **(C)** Half-maximal effective concentration (EC_50_) value calculated based on cell viability results **(D)** HT-29 cells were pre-treated with various concentrations of celastrol before TSZ stimulation for 12 h. LDH release was measured using an LDH assay kit **(E)** Representative calcein-AM/PI staining images of HT-29 cells treated with celastrol for 12 h under different conditions. ^**^ *P* < 0.01, ^*^ *P* < 0.05 versus the TSZ group; ^##^ *P* < 0.01, ^#^ *P* < 0.05 versus the CON group. *N* = 3 per group.

The original version of this article has been updated.

